# Estimation of lifetime costs for patients receiving a transplant: the case of liver transplantation related to hepatitis B in Italy

**DOI:** 10.3389/fpubh.2024.1328782

**Published:** 2024-07-03

**Authors:** Alfredo Marzano, Beatrice Canali, Luciano De Carlis, Paolo De Simone, Francesca Fiorentino, Maria Rendina, Chiara Vassallo, Stefano Fagiuoli

**Affiliations:** ^1^Gastroenterology and Hepatology Unit, San Giovanni Battista Hospital, Turin, Italy; ^2^Real World Solutions, IQVIA Solutions Italy S.R.L., Milan, Italy; ^3^Department of General Surgery and Transplantation, Niguarda Hospital, Milan, Italy; ^4^Hepatobiliary Surgery and Liver Transplantation Unit, University of Pisa Medical School Hospital, Pisa, Italy; ^5^Gastroenterology Department of Emergency and Organ Transplantation, University Hospital Policlinico di Bari, Bari, Italy; ^6^Department of Medicine, University of Milan Bicocca and Gastroenterology Hepatology and Transplantation Unit, Papa Giovanni XXIII Hospital, Bergamo, Italy

**Keywords:** hepatitis B, immunoglobulin, liver transplant, cost analysis, cost of illness, Italy

## Abstract

**Introduction:**

In Italy, post-liver transplant (LT) hepatitis B virus (HBV) reinfection prophylaxis is frequently based on a combined regimen of anti-HBV immunoglobulin (HBIG) and oral antivirals. However, little information is available at the national level on the cost of LT and the contribution of HBV prophylaxis. This study aimed to quantify the direct healthcare cost for adult patients undergoing LT for HBV-related disease over a lifetime horizon and from the perspective of a National Healthcare Service.

**Methods:**

A pharmaco-economic model was implemented with a 4-tiered approach consisting of 1) preliminary literature research to define the research question; 2) pragmatic literature review to retrieve existing information and inform the model; 3) micro-simulated patient cycles; and 4) validation from a panel of national experts.

**Results:**

The average lifetime healthcare cost of LT for HBV-related disease was €395,986. The greatest cost drivers were post-transplant end-stage renal failure (31.9% of the total), immunosuppression (20.6%), and acute transplant phase (15.8%). HBV reinfection prophylaxis with HBIG and antivirals accounted for 12.4% and 6.4% of the total cost, respectively; however, lifetime HBIG prophylaxis was only associated with a 6.6% increase (~€422 k). Various sensitivity analyses have shown that discount rates have the greatest impact on total costs.

**Conclusion:**

This analysis showed that the burden of LT due to HBV is not only clinical but also economic.

## Introduction

Hepatitis B virus (HBV) infection is the most common chronic viral disease worldwide, with an estimated prevalence of 4.1% in 2019 (1). According to the most recent estimates (1, 2), approximately 425,000 people were chronically infected in Italy in 2014. Over the last 30 years, the epidemiological and clinical scenarios of both the general population and liver transplant patients have radically changed in most Western countries owing to the introduction of vaccination and nucleos(t)ide analogs (NAs) in the former and the use of anti-hepatitis B immunoglobulin (HBIG) in the latter. Vaccination (1) has resulted in a significant reduction in the incidence of HBV infection [from approximately 10 cases/100,000 inhabitants in the 1990s to 1 case/100,000 in 2011 in Italy (3)], while HBIG and antiviral drugs have improved the outcome of both chronic HBV patients and liver transplant recipients, resulting in a 5-year survival rate above 80% versus 45% prior to HBIG introduction (4).

Despite these advancements, HBV infection remains a major public health burden (5) and a major risk factor for liver cirrhosis, hepatocellular carcinoma (3), and liver failure (6). Liver transplantation (LT) is the best therapeutic option for HBV-related end-stage liver disease but is associated with early and long-term complications, immunosuppression-related comorbidities, and high socio-economic costs (7).

Post-transplant HBV prophylaxis with a combination of HBIG and NA tailored to patient-, transplant-, and virus-related risk factors is crucial to favorable long-term results, but concerns about the cost of HBIG and the availability of high-barrier NA have gradually reduced the use, dose, and duration of immunoglobulin in the last few years (5). However, information on the overall economic burden of LT in general, and post-transplant HBV prophylaxis in particular, is still scarce in Europe (8). To fill this gap, we performed the present study to quantify the direct healthcare costs for patients undergoing LT for HBV infection over a lifetime horizon and from the perspective of the Italian National Healthcare System (NHS).

## Materials and methods

To estimate the direct lifetime healthcare cost of LT for HBV-related liver disease, we conducted an analysis using a multi-step approach, in line with the methodology reported by the National Institute for Health and Care Excellence (NICE) (9). Initially, a targeted literature review was performed to conceptualize the research question and identify the key components needed to implement a lifetime model for HBV patients undergoing LT. A pragmatic literature review of the available evidence on LT in Europe was then performed to retrieve the necessary information to inform the model. Finally, the model was conceptualized and implemented using the statistical program *Stata/MP,* version 14.0. The model structure and inputs were validated by two Italian key opinion leaders (KOLs), while resource consumption and costs were calibrated with the support of three additional KOLs. The model was then adjusted and finalized following the discussions with the five clinical experts.

### Problem conceptualization and pragmatic literature review

**Figure 1:**
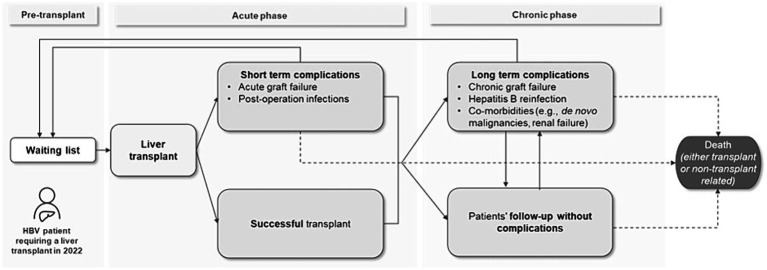
Preliminary patient flow of the model. HBV, Hepatitis B Virus.

To assess model feasibility, the research question was focused on through preliminary literature research, and the patient flow was outlined based on published evidence (4, 10). The initial flow is divided into three phases: pre-transplant, post-transplant acute phase and post-transplant chronic phase (Figure 1). The patients begin the flow entering the waiting list for liver transplantation and after an average waiting time they undergo transplantation, successfully or with acute complications. Finally, they enter the post-transplant chronic phases, where they might have a post-LT follow-up without any event or incur in chronic complications. At any point, patients might undergo re-transplantation (re-LT) or die, due to transplant- or non-transplant-related causes. The initial flow’s structure was discussed and validated with two KOLs.

After conceptualizing the problem and validating the initial flow, a pragmatic review of the scientific literature was conducted according to the PRISMA guidelines to retrieve the available information and define the final model structure. Information retrieved from literature included probabilities of events’ occurrence, time to transplant, time to complications, time to death, and resource consumption for each phase. The search was conducted in January 2022 by querying the online databases Medline (Pubmed), Cochrane Library, and Embase with a combination of eight different research strings, and it was focused on papers published for the European context from January 1st, 2010, onwards (Table 1). Inclusion, selection criteria, and search terms were discussed and validated with two KOLs. Search terms referred to the patient flow phases identified during the problem conceptualization phase in terms of events, patient management, and cost analyses. Detailed information on the PRISMA flow diagram and the resulting sources is provided in the Supplementary material.

**Table 1:** Inclusion and preferred selection criteria of the pragmatic literature review.

	Inclusion criteria	Preferred selection criteria
Population	Patients who received a liver transplant	Adult patients who received a liver transplant due to CHBV infection, without acute liver failure or other co-infections
Time frame	From 2010 onwards	From 2015 onwards
Geography	Europe	Italy
Study type	Pragmatic reviews and meta-analysis Randomized control trials Cohort studies Prospective studies Retrospective studies	Pragmatic reviews and meta-analysis Randomized control trials Cohort studies

CHBV, Chronic Hepatitis B Virus.

### Model conceptualization

**Figure 2:**
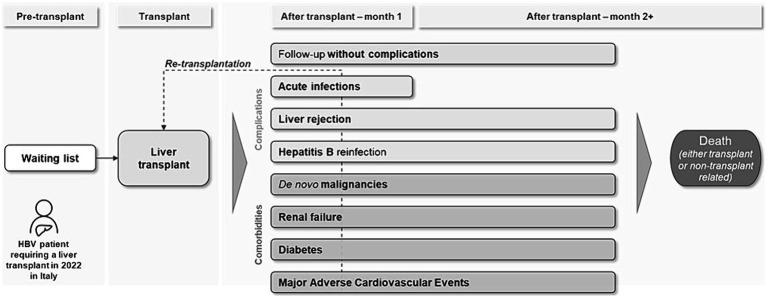
Model structure. HBV, Hepatitis B Virus.

Based on the information retrieved from the pragmatic literature review, the final model structure was conceptualized (Figure 2). The patient enters the model in the waiting list and undergoes transplantation after an average waiting time, retrieved from published data reports (11–13). In the post-transplant period, patients are either followed up regularly with routine examinations and HBV prophylactic treatment, or they experience complications (postoperative infections, graft rejection, HBV reinfection) and comorbidities [*de novo* malignancies, renal failure, diabetes, major adverse cardiovascular events (MACEs)].^1^ At any time, patients may undergo re-transplantation (re-LT)^2^ or die due to either transplant- or non-transplant-related causes.

Due to the complexity of patient follow-up, a microsimulation approach was chosen to build the model, since it is considered more appropriate for complex chronic diseases where patient-level information is relevant (14). Microsimulations allow the replication of the healthcare trajectory of individual patients (15), which can be affected by more than one complication/comorbidity at a time, and to keep track of each patient’s individual history. Patients enter the model in the initial state and proceed individually through various transition states based on the probabilities of transition and are subject to events with a time-varying probability of occurrence.

The model simulated 10,000 adult patients who underwent LT for HBV-related disease and were followed up until death. It consisted of 12 one-month cycles in the early (i.e.,1 year) post-transplant period followed by 44 one-year cycles, for a maximum time horizon of 45 years [which corresponds to a projected 100-year life span assuming a mean age of 55 years at wait listing (11)]. Costs were discounted at the present value using an annual discount rate of 3%, according to the Italian guidelines for economic evaluations (16).

The model starts in the waiting list and assumes that all patients are transplanted, since the study aims to estimate the costs associated with LT from the pre-transplant through the post-transplant phase. After LT, patients may experience complications and comorbidities, according to the estimated probabilities of occurrence. The model assumes that complications are independent of each other and that patients may only undergo re-LT once during their lifetime. If a patient develops any long-term complication in cycle t, the complication will persist over time (i.e., from cycle t + 1 onwards), and the cost associated with the events is quantified and assigned to the patient.

At the end of each cycle, patients may die according to general LT population survival curves or comorbidities-specific curves, consistently with literature data (17). Finally, if, at any time point, the death probability was lower than that of the sex- and age-adjusted Italian population, the latter was applied.

The direct healthcare lifetime cost of an average patient undergoing LT for HBV-related disease was calculated by running 1,000 simulations of the model for 10,000 patients, to allow for the convergence of in-sample standard errors toward zero (see the Supplementary material for more details). Results were extracted from each simulation in terms of average total lifetime cost, average lifetime cost by cost component, average cost by transplantation phase, and probability of occurrence of each complication and comorbidity at different model cycles. The average total lifetime cost for one patient was then multiplied by the estimated eligible population in Italy of 233 patients (18, 19).^3^

### Model input parameters

#### Incidence curves

To assess probability of occurrence of comorbidities and complications in each cycle of the model, incidence curves related to model events were retrieved from the literature, except for postoperative infection and re-transplantation, which were modeled by the authors using different probability distributions based on literature data (13, 20–22). The curves retrieved from the literature were extrapolated beyond their original follow-up period to cover the entire patient’s life, using the fittest parametric model. The choice of the parametric model was made on a case-by-case basis on both visual inspection and statistical criteria (see Supplementary material for more details) and was later validated by two KOLs. In the final model, some of the original curves were adjusted to better mirror Italian clinical practice, according to inputs from the KOLs. Table 2 provides an overview of the sources and the models used for each incidence curve, as well as the assumptions made in terms of curve adjustment following KOLs’ validation.

**Table 2:** Resume of parametric models used for incidence curves of model events.

Comorbidity/Complication	Parametric model	Assumptions	Source
Post-operative infections	Gamma^a^	–	(13, 20)
Liver rejection	Hazard 1 knot	–	(17)
Re-LT	Gompertz^b^	–	(21, 22)
HBV recurrence	Gompertz	Cumulative incidence curve from the source was assumed constant after year 2	(23)
*De novo* malignancies	Gompertz	–	(24)
Renal failure	Gompertz	–	(61)^c^
Diabetes	Gompertz	–	(25)
MACEs	Gompertz	Cumulative incidence curve from the source was adjusted considering a relative risk of incidence of 0.5, since among liver transplantation patients, those infected with HBV have the lowest relative risk of MACEs among all etiologies	(62)^d^

Re-LT, Re-transplantation; HBV, Hepatitis B; MACEs, Major Adverse Cardiovascular Events. (a) Curve not identified through the pragmatic literature review (PLR): the Gamma distribution was constructed by the authors around the average probability of infection retrieved from the literature (13, 20); (b) Curve not identified through the PLR: the Gompertz distribution was constructed by the authors based on the cumulative 1-year and lifetime incidence of re-LT retrieved from the literature (21, 22); (c) Source suggested by KOLs, as it represented the Italian scenario better than the source originally selected through the PLR; (d) Source identified through additional research and later validated with KOLs, since at the time the PLR was conducted, MACEs were not part of the patient flow.

#### Survival curves

Mortality in each cycle was modeled by extrapolating complication- and comorbidity-specific survival curves from the literature. Similarly to incidence curves, the choice of the fittest parametric model for survival curve extrapolations was assessed through visual inspection and statistical criteria (see Supplementary material for more details) and was later validated and adjusted with two KOLs (Table 3). In particular, event-specific mortality for patients who developed post-operative infections, diabetes, and HBV recurrence was assumed equal to the general mortality for patients undergoing transplantation due to HBV retrieved from the literature (17), while mortality for patients who develop liver rejection and renal failure was computed by adjusting the same general mortality curve using relative risk factors. Patients who develop *de novo* tumors or MACEs and patients who are subject to re-transplantation follow their respective event-specific mortality. Finally, patients who persist in post-LT follow-up without any comorbidities or complications are subject to their own mortality curve. This curve was computed at each cycle by considering the weighted sum of all other curves applied in the same cycle, aiming to achieve a general mortality for patients who undergo LT due to HBV that aligns with the literature (17).

**Table 3:** Resume of parametric models used for survival curves of model events.

Comorbidity/Complication	Parametric model	Assumptions	Source
Post-operative infections	Hazard 2 knots	Assumed equal to the survival curve for the general population of patients undergoing transplantation due to HBV	(17)
Liver rejection	Hazard 2 knots, adjusted	The survival curve from the source was adjusted considering a relative risk of survival of 0.78 for patients with liver rejection	(17, 26)
Re-LT	Hazard 2 knots	-	(27)
HBV recurrence	Hazard 2 knots	Assumed equal to the survival curve for the general population of patients undergoing transplantation due to HBV	(17)
*De novo* malignancies	Hazard 2 knots	-	(28)
Renal failure	Hazard 2 knots, adjusted	The survival curve from the source was adjusted considering a relative risk of survival of 0.9 for patients with renal failure	(17)
Diabetes	Hazard 2 knots	Assumed equal to the survival curve for the general population of patients undergoing transplantation due to HBV	(17)
MACEs	Hazard 2 knots	-	(29)

Re-LT, Re-transplantation; HBV, Hepatitis B; MACEs, Major Adverse Cardiovascular Events.

#### Resource consumption and costs

Unit costs were either per event or annual. The formers were assigned only once to the corresponding cycle of occurrence; the latter were assigned to the patient annually, from the date of complication/comorbidity until death, except for *de novo* malignancies and HBIG prophylaxis. Consistent with other modeling approaches (30), the annual costs of *de novo* malignancies started in the incident year throughout the five subsequent years.

The unit cost of LT and postoperative infections was estimated based on 2022 national tariffs (31). The unit costs of the waiting list, complications, follow-up, and prophylactic treatment were calculated using a micro-costing approach, considering the relevant resource consumption of visits and exams, hospitalization episodes, and pharmacological treatment for each component. Resource consumption was retrieved from national guidelines (32), summary of product characteristics of relevant drugs (33), and published literature (34–36) and was later validated with the five KOLs. The unit costs of visits, exams, and hospitalizations were retrieved from national tariffs (31, 37), while the cost of drugs was calculated from the ex-factory prices net of mandatory discounts, as reported in the Italian Official Journal (38). Finally, the costs of comorbidities were retrieved from Italian literature (31, 39–55) and inflated to 2022.

Annual costs of HBIG were imputed considering their variable duration and were interrupted in the event of HBV recurrence (56). The duration of HBIG prophylaxis was modeled considering the consensus gathered from the Italian IMMUNOHBs expert meeting in 2020, during which 24 experts from the Italian liver transplantation community agreed on post-LT prophylaxis protocols based on the available evidence and clinical practice (36). The resource consumption and unit cost details are outlined in Tables 4, 5, respectively. Table 6 presents the cost values used to inform the model, expressed in euros (€) for the year 2022.

**Table 4:** Resource consumption considered for micro-costing.

Exams^a^		Hospitalization		Treatment	
Description (32)	No. of patients/Frequency (32)	Description (32)	No. of patients/Frequency (32)	Drug (32)	No. of patients/Daily posology^b^ (33)
Waiting list					
Alanine Aminotransferase; Albumin; Bilirubin; Blood count; Cholesterol; Creatinine; Creatinine clearance; Gamma Glutamyl Transpeptidase; HBV DNA-polymerase; HBV HBsAg antibodies; Prothrombin time; Sodium; Triglycerides	100% of patients/every 2.5 months	Stomach interventions	50% of patients/1.5 episodes	Entecavir	60% of patients/ 1 mg
Alpha-1 fetoprotein; Glucose; Urine exam	100% of patients/every 6 months	Cirrhosis and alcoholic hepatitis	50% of patients/3 episodes	Tenofovir disoproxil	20% of patients/245 mg
Ultrasound	50% of patients/every 6 months			Tenofovir alafenamide	20% of patients/ 25 mg
CT scan	50% of patients/every 3 months			Potassium Canrenoate	55% of patients/125 mg
				Furosemide	55% of patients/100 mg
				Propranolol	35% of patients/240 mg
				Carvedilol	35% of patients/ 50 mg
				Rifaximin (Tixteller)	50% of patients/1,100 mg
				Rifaximin (Normix)	50% of patients/800 mg
				Albumin	15% of patients/250 mg/kg
				Pantoprazole	100% of patients/40 mg
				Lansoprazole	100% of patients/30 mg
				Gliclazide	20% of patients/75 mg
Prophylaxis with immunosuppressors					
–	–	–	–	Tacrolimus	70% of patients/0.15 mg/kg
				Everolimus	10% of patients/2 mg
				Reduced-dose TAC + Everolimus	10% of patients/0.08 mg/kg^c^ + 2 mg
				Cyclosporine	10% of patients/ 4 mg/kg
				Mycophenolic acid	75% of patients/1,000 mg
Prophylaxis with HIBGs (36)					
–	–	–	–	Human Ig SC (low-risk patients)	46% of patients/ 33 IU
				Human Ig IM *(low-risk patients)*	35% of patients/ 37 IU
				Human Ig SC *(high-risk patients)*	12% of patients/ 40 IU
				Human Ig IM *(high-risk patients)*	9% of patients/ 43 IU
Prophylaxis with antivirals					
–	–	–	–	Entecavir	60% of patients/ 1 mg
				Tenofovir disoproxil	20% of patients/ 245 mg
				Tenofovir alafenamide	20% of patients/ 25 mg
Follow-up					
Alanine Aminotransferase; Albumin; Bilirubin; Blood count; Cholesterol; Creatinine; Creatinine clearance; Fecal occult blood; Gamma Glutamyl Transpeptidase; Glucose; Immunosuppressor trough level; HBV HBsAg antigen; HBV HBsAg antibodies; Prothrombin time; Sodium; Triglycerides; Urine exam	100% of patients/4 times/year^d^	–	–	–	–
Alpha-1 fetoprotein	50% of patients/ 4 times/ year^d^				
Liver rejection					
Alanine Aminotransferase; Albumin; Alkaline phosphatase; Alkaline phosphatase bone isoenzyme; Aspartate Aminotransferase; Bilirubin; Blood count; Creatinine; Gamma Glutamyl Transpeptidase; Glucose; Immunosuppressor trough level; Prothrombin time; Sodium CT angiography; CT scan; Ultrasound	150% of patients with liver rejection/ per liver rejection episode	Cirrhosis and alcoholic hepatitis	5% of patients/ 1 episode	Methylprednisolone	100% of patients with liver rejection/ 1,000 mg
HBV recurrence					
HBV DNA-polymerases	100% of patients with HBV recurrence/per recurrence episode	–	–	–	–

HBV, Hepatitis B virus; HBsAg, Hepatitis B surface Antigen; CT, Computerized tomography; HBIGs, Hepatitis B immunoglobulins; Ig, Immunoglobulin; SC, Subcutaneous; IM, Intramuscular; IU, International Units. (a) In addition to the set of exams listed, for each cost component one venous blood Research Topic and one visit with a specialistic practitioner is considered; (b) When required, an average weight of 70 kg was considered, in order with Italian guidelines for economic modeling (16); (c) To determine the posology of the reduced-dose tacrolimus, the standard posology of tacrolimus from the SmPC was decreased proportionally to the reduction of the trough level of tacrolimus in the blood in the reduced-TAC + everolimus arm with respect to the full-TAC arm in De Simone et al. (57); (d) Frequency refers to the 2+ year post-LT. In the first year post-LT visits and exams occur with a frequency of 7 times/ year.

**Table 5:** Unit costs considered for micro-costing.

Item description	Unit cost	Source (31, 37, 38)
Exams and visits		
Alanine aminotransferase	€1.00	Tariffario ambulatoriale 2013: code 90.04.5
Albumin	€1.42	Tariffario ambulatoriale 2013: code 90.05.1
Alkaline phosphatase	€1.04	Tariffario ambulatoriale 2013: code 90.23.5
Alkaline phosphatase bone isoenzyme	€12.33	Tariffario ambulatoriale 2013: code 90.24.1
Alpha-1 fetoprotein	€7.40	Tariffario ambulatoriale 2013: code 90.05.5
Aspartate Aminotransferase	€1.04	Tariffario ambulatoriale 2013: code 90.09.2
Bilirubin	€1.13	Tariffario ambulatoriale 2013: code 90.10.4
Blood count	€3.17	Tariffario ambulatoriale 2013: code 90.62.2
Cholesterol	€1.04	Tariffario ambulatoriale 2013: code 90.14.3
Creatinine	€1.13	Tariffario ambulatoriale 2013: code 90.16.3
Creatinine clearance	€1.60	Tariffario ambulatoriale 2013: code 90.16.4
Fecal occult blood	€3.52	Tariffario ambulatoriale 2013: code 90.21.4
Gamma Glutamyl Transpeptidase	€1.13	Tariffario ambulatoriale 2013: code 90.25.5
Glucose	€2.38	Tariffario ambulatoriale 2013: code 90.26.4
HBV DNA-polymerase	€23.34	Tariffario ambulatoriale 2013: code 91.19.2
HBV HBsAg antibodies	€10.01	Tariffario ambulatoriale 2013: code 91.18.3
HBV HBsAg antigen	€10.01	Tariffario ambulatoriale 2013: code 91.18.4
Immunosuppressor trough level^a^	€14.64	Tariffario ambulatoriale 2013: code 90.13.2
Prothrombin time	€2.85	Tariffario ambulatoriale 2013: code 90.75.4
Sodium	€1.02	Tariffario ambulatoriale 2013: code 90.40.4
Triglycerides	€1.17	Tariffario ambulatoriale 2013: code 90.43.2
Urine exam	€2.17	Tariffario ambulatoriale 2013: code 90.44.3
CT angiography	€158.04	Tariffario ambulatoriale 2013: code 88.01.6
CT scan	€103.68	Tariffario ambulatoriale 2013: code 88.01.5
Ultrasound	€60.43	Tariffario ambulatoriale 2013: code 88.76.1
Specialistic visit^b^	€20.66	Tariffario ambulatoriale 2013: code 89.7
Venous blood Research Topic	€2.58	Tariffario ambulatoriale 2013: code 91.49.2
Inpatient hospitalization		
Cirrhosis	€4,013.00	Tariffario prestazioni per acuti 2013: DRG 202
Hepatocellular carcinoma	€6,566.00	Tariffario prestazioni per acuti 2013: DRG 155
Drugs^c^		
Entecavir	€319.91	Italian Official Journal
Tenofovir disoproxil	€1.63	Italian Official Journal
Tenofovir alafenamide	€39.11	Italian Official Journal
Potassium Canrenoate	€0.12	Italian Official Journal
Furosemide	€0.10	Italian Official Journal
Propranolol	€0.08	Italian Official Journal
Carvedilol	€1.10	Italian Official Journal
Rifaximin (Tixteller)	€0.50	Italian Official Journal
Rifaximin (Normix)	€0.22	Italian Official Journal
Albumin	€0.27	Italian Official Journal
Pantoprazole	€0.63	Italian Official Journal
Lansoprazole	€0.62	Italian Official Journal
Gliclazide	€0.18	Italian Official Journal
Tacrolimus	€86.46	Italian Official Journal
Everolimus	€694.02	Italian Official Journal
Cyclosporine	€2.55	Italian Official Journal
Mycophenolic acid	€0.64	Italian Official Journal
Human immunoglobulin SC formulation	€44.60	Italian Official Journal
Human immunoglobulin IM formulation	€32.72	Italian Official Journal
Methylprednisolone	€1.40	Italian Official Journal

HBV, Hepatitis B Virus; HBsAg, Hepatitis B surface Antigen; CT, Computerized Tomography; SC, Subcutaneous; IM, Intramuscular. (a) Assumed equal to cost of cyclosporine trough level for all immunosuppressor drugs; (b) Assumed equal to cost of general visit; (c) Reported costs reflect ex-factory prices net of temporary discounts for 100 mg or 100 IU of drug.

**Table 6:** Values used to inform the model, by cost component.

Cost component	Value	Type of cost
LT costs	Waiting list^a,b^	€ 14,915 (31, 37, 38)	Event-based
	Liver transplantation	€ 62,648 (31)	Event-based
Prophylaxis and follow-up costs	Prophylaxis with immunosuppressors	€ 5,503 (38)	Annual
	Prophylaxis with antivirals	€ 1,707 (38)	Annual
	Prophylaxis with HBIG^d,e^	€ 5,191 (38)	Annual
	Follow-up – first year^f^	€ 554 (37)	Annual
	Follow-up – second year+	€ 345 (37)	Annual
Complications costs	Post-operative infections	€ 9,163 (31)	Event-based
	Liver rejection^c^	€ 743 (31, 37, 38)	Event-based
	Re-transplantation	€ 62,648 (31)	Event-based
	HBV recurrence^g^	€ 44 (37)	Event-based
Comorbidities costs	*De novo* malignancies^h^	€ 8,081 (39–48)	Annual
	Renal failure – management^i^	€ 33,335 (49, 50, 61)	Annual
	Renal failure – transplantation^j^	€ 55,943 (51)	Event-based
	Diabetes	€ 3,208 (53)	Annual
	MACEs – event^k^	€ 5,820 (31, 54, 55)	Event-based
	MACEs – follow-up^l^	€ 3,039 (31, 52, 54, 55, 74)	Annual

LT, Liver Transplant; HBV, Hepatitis B; HBIG, Hepatitis B Immunoglobulins; MACEs, Major Adverse Cardiovascular Events. (a) For an average waiting time of 255 days, in coherence with the literature (58, 59); (b) Of which €450 of visits and exams, €10,944 of hospitalization, and €3,521 of pharmacological treatment; (c) Of which €500 of visits and exams, €201 of hospitalization, and €42 of pharmacological treatment; (d) Weighted average between cost of intramuscular formulation and subcutaneous formulation for high-risk and low-risk patients; (e) Cost of initial HBIG intravenous administration of 10,000 IU/week in the hepatic phase (6) is not considered, as it is covered by the DRG tariff for liver transplantation since patients are discharged after an average of 28 days (13, 60); (f) First month follow-up cost is not considered, as it is assumed to be covered by the DRG tariff for liver transplantation since patients are discharged after an average of 28 days (13, 60); (g) Includes cost of HBV DNA-polymerases and specialistic visit; (h) Weighted average between annual costs of skin non-melanoma, blood cancer, gastrointestinal cancer, head and neck cancer, bronchus and lungs cancer, bladder cancer, skin melanoma, thyroid gland cancer, kidney cancer, and breast cancer, using as weights the respective frequencies in LT patients retrieved from Taborelli et al. (24); (i) Weighted average between annual cost of chronic kidney disease (CKD) stages 4–5 (50), annual cost of renal failure with dialysis (49), and annual cost of post-renal transplantation follow-up (51) using as weights, respectively, the percentage of patients with renal failure who are in stage 4–5 of CKD, the percentage of patients with renal failure who are in dialysis, and the percentage of patients with renal failure who underwent renal transplantation, retrieved from Ojo et al. (61); (j) Cost of renal transplantation from (51) will be multiplied by the percentage of patients with renal failure who undergo renal transplantation, retrieved from Ojo et al., 2003 (61); (k) Weighted average between cost of acute coronary syndrome, angina, congestive heart failure, stroke, arrhythmia, and peripheral artery disease using as weights the respective frequencies in LT patients retrieved from Albeldawi et al. (62); (l) Weighted average between cost of follow-up for acute coronary syndrome, angina, and stroke, using as weights the respective frequencies in LT patients retrieved from Albeldawi et al. (62).

### Deterministic sensitivity analyses

A set of deterministic sensitivity analyses was conducted based on the uncertainties of some of the model parameters. Regarding resource consumption, different HBIG and immunosuppressive regimens were considered. For HBIG treatment, based on previous discussions about the optimal duration of treatment in clinical practice (36, 56), two alternative scenarios were explored: 1-year prophylaxis versus lifetime administration in order to assess variation of costs resulting from the two extreme cases. These scenarios were modeled by assigning related costs for the respective durations, without varying the effect of prophylaxis on health outcomes since no published evidence relating prophylaxis duration and risk health outcomes was found (6). For immunosuppression, posology variations of −30% and −50% were explored owing to drug dose modifications in the early post-transplant period (35). Regarding complications/comorbidities, the incidence of end-stage renal failure derived from Ojo et al. (61) was decreased by 30% due to changes observed in clinical practice over the last two decades. Furthermore, to address possible uncertainties which might arise from the choice of the parametric models used to extrapolate incident and survival curves, two sensitivity analyses were conducted on the curve informing the cost items with the highest impact on the model results (i.e., overall survival and renal failure incidence). In particular, overall survival was modeled using the odds 1-knots spline model, while renal failure incidence was modeled after incidence using the gamma distribution. The first analysis allows to assess results in the best-case scenario, i.e., the scenario with the lowest mortality, while the second in the worst-case scenario, i.e., the scenario with the highest renal failure incidence.

Two sensitivity analyses were conducted to assess the impact of different unit costs. First, the weighted average price^4^ of all drugs included in the model was used instead of the ex-factory price net of mandatory discounts (38). Second, variations of ±15% were considered for cost components of LT (cost of waiting list and cost of surgery), complications (cost of postoperative infections, liver rejection, and HBV recurrence), and comorbidities (cost of *de novo* malignancies, renal failure, diabetes, and MACEs), to address concerns of possible underestimation or overestimation of cost components. Finally, two scenarios were explored by considering alternative discount rates (0% versus 5%) in line with the Italian guidelines for economic evaluations (16).

## Results

**Figure 3:**
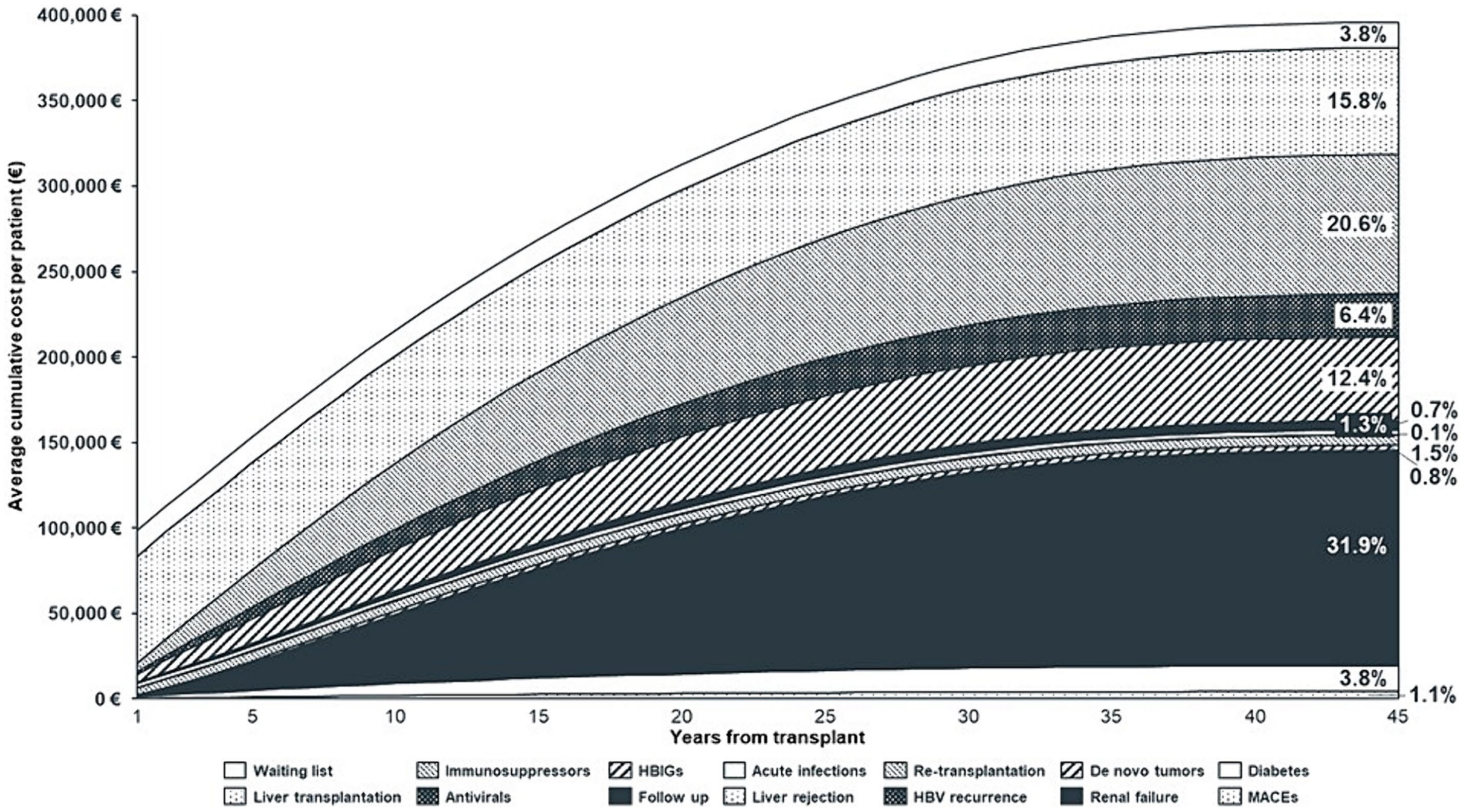
Average cumulative cost per patient, by year and cost component (Discounted Values). HBIGs, Hepatitis B Immunoglobulins; HBV, Hepatitis B Virus; MACEs, Major Adverse Cardiovascular Events.

The main results of the analysis in terms of costs are shown in Table 7 and Figure 3. The average lifetime direct healthcare cost for an adult recipient undergoing LT for HBV-related disease in Italy was estimated to be €395,986, varying between € 386,368 and € 404,745 among the different iterations (Table 7). Considering the incidence estimates derived from the literature (Table 2) and the values of cost components used to populate the model (Table 6), the greatest cost driver was post-transplant end-stage renal failure, accounting for 31.9% (~€126 k) of the total cost, followed by the cost of immunosuppression (20.6%, ~€81 k) and cost of liver transplantation (15.8%, ~€63 k). HBV prophylaxis with HBIG and antivirals accounted for 12.4% (~€49 k) and 6.4% (~€25 k) of the total cost, respectively. Finally, the cost of the waiting list accounted for 3.8% (~€15 k) of the total cost, while follow-up, complications, and comorbidities other than renal failure were residual.

**Table 7:** Average cost per patient, by cost component (discounted values).

	Average cost per patient^a^ (min-max)	% of total cost per patient
Total average cost per patient	€ 395,986^b^ (€ 386,368-€ 404,745)	100%^c^
LT costs	Waiting list	€ 14,915 (−)^d^	3.8%
	Liver transplantation	€ 62,648 (−)^d^	15.8%
Prophylaxis and follow-up costs	Prophylaxis with immunosuppressors	€ 81,472 (€ 79,505-€ 83,315)	20.6%
	Prophylaxis with antivirals	€ 25,267 (€ 24,657-€ 25,839)	6.4%
	Prophylaxis with HBIGs	€ 48,994 (€ 47,038-€ 50,676)	12.4%
	Follow-up	€ 5,297 (€ 5,172-€ 5,415)	1.3%
Complications costs	Post-operative infections	€ 2,821 (€ 2,696-€ 2,977)	0.7%
	Liver rejection	€ 234 (€ 223-€ 243)	0.1%
	Re-transplantation	€ 5,785 (€ 5,041-€ 6,448)	1.5%
	HBV recurrence	€ 1 (€ 0.91-€ 1.32)	0.0%
Comorbidities costs	*De novo* malignancies	€ 3,135 (€ 2,864-€ 3,406)	0.8%
	Renal failure	€ 126,163 (€ 119,529-€ 134,117)	31.9%
	Diabetes	€ 15,016 (€ 14,097-€ 16,038)	3.8%
	MACEs	€ 4,236 (€ 3,670-€ 4,715)	1.1%

LT, Liver Transplant; HBV, Hepatitis B; HBIGs, Hepatitis B Immunoglobulins; MACEs, Major Adverse Cardiovascular Events. (a) Average cost per patient of each component is reflective of the incidence (Table 2) and cost (Table 6) used to populate the model; (b) Sum of cost components might not add to total, due to rounding; (c) Sum of percentages might not add to 100%, due to rounding; (d) Variation not reported because these costs are sustained by all patients in all iterations.

Regarding the liver transplantation phases, the cost of the waiting list and transplantation was €77,563. In the first-year post-transplantation, when follow-up visits are more frequent, HBV prophylaxis doses are higher (32, 35, 36) and complications and comorbidities – such as infections, re-LT, HBV recurrence, and diabetes – are more likely to occur (13, 20–23, 25); the average annual cost per patient was €20,818. From the second year onwards, this cost decreased to an average of €6,764/patient per year.

Figure 3 illustrates the cumulative cost of LT and shows that the annual increase in costs declines over time. This was due to: (1) some costs being on–off at the beginning of the model (waiting list and transplant), (2) higher incidence of complications/comorbidities in the early post-transplant period, (3) higher resource consumption in the early years following transplantation (follow-up and prophylaxis), (4) patients exiting the model due to death, and (5) application of the discount rate.

Considering the eligible population, that is, patients who underwent liver transplantation in Italy due to HBV in 2022 (233 patients) (see foot note 3) (18, 19), the discounted lifetime economic burden for the Italian NHS related to HBV-patients was €92.3 million.

Table 8 reports the probability of occurrence of complications predicted by the model. The most frequent complications on a lifetime horizon were liver rejection, renal failure, and diabetes, all reaching estimates of cumulative incidence above 30%. Focusing on short-term comorbidities and complication, re-transplantation emerges as one of the most frequent complications (6.5%). Overall, patients in the model had an average life expectancy of 22 years, with a 1-year, 3-year, and 5-year probability of survival of 86.1, 82.7, and 80.1%, respectively.

**Table 8:** Cumulative probability of occurrence of events in the model.

Event	1-year	3-year	5-year	10-year	Lifetime*
Post-operative infections	30.89%	–	–	–	–
Liver rejection	13.02%	19.86%	23.18%	27.84%	38.76%
Re-transplantation	6.45%	9.18%	9.49%	9.54%	9.54%
HBV recurrence	1.35%	2.61%	–	–	–
De novo malignancies	1.24%	3.34%	5.00%	7.84%	11.92%
Renal failure	4.8%	12.11%	17.19%	24.59%	32.56%
Diabetes	31.49%	31.51%	31.51%	31.51%	31.51%
MACEs	2.18%	4.97%	6.51%	8.08%	8.67%
Death from any cause	13.92%	17.30%	19.94%	27.02%	100.00%

HBV, Hepatitis-B Virus; MACEs, Major Adverse Cardiovascular Events. *Corresponding to a model time-horizon of 45 years.

These estimates are aligned with published data from Italian reports and observational studies. For example, Angelico et al. (58) report a re-transplantation rate of 6.1% at 18-month follow-up, while the Italian national guidelines on LT (32) report an incidence rate between 3 and 15% for *de novo* tumors and between 19 and 30% for renal failure. Similarly, an official report from the Italian National Transplant Center evaluating activities related to LT in Italy provides patients’ and grafts’ survival estimates comparable to our study (63).

### Deterministic sensitivity analyses

**Figure 4:**
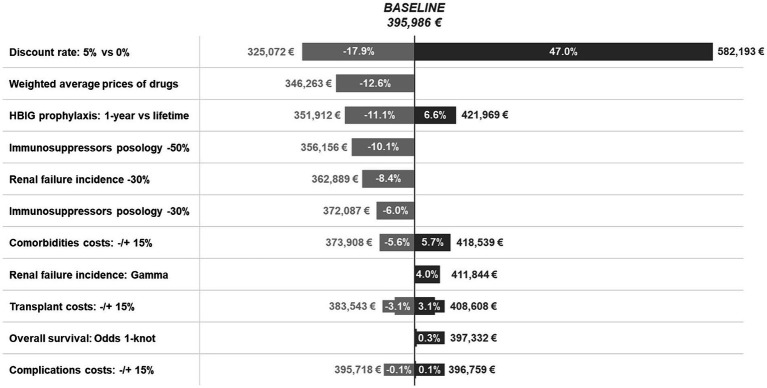
Deterministic sensitivity analyses.

The results of the deterministic sensitivity analyses are shown in Figure 4. According to the different scenarios, the cost of LT for HBV-related disease ranged from €325,072 to €582,193 per patient compared to the baseline scenario (€395,986/patient). Discount rates had the greatest impact on cost, increasing by 47.0% (~€582 k) for a 0% discount rate.

As for drugs, when the weighted average price was considered instead of the official ex-factory price, the average total lifetime cost of LT was €346,263 per patient, namely 12.6% lower than baseline, due to a 24.9% reduction in the annual cost of immunosuppression and a 26.3% and 61.8% decrease in the annual cost of HIBG and antivirals, respectively.

One-year HBIG prophylaxis resulted in an 11.1% decrease (~€352 k) in the total cost, while lifetime prophylaxis was associated with a 6.6% increase (~€422 k). Accordingly, a 30 and 50% reduction in immunosuppressant posology 3 months post-transplantation resulted in a 6.0% (~€372 k) and 10.1% (~€356 k) decrease in total cost, respectively.

Variations of −/+ 15% for cost components related to comorbidities, liver transplantation, and complications led to variations from the baseline results of −/+ 5.7% (~€374 k; ~€419 k), −/+ 3.1% (~€384 k; ~€409 k), and −/+ 0.1% (~€396 k; €397 k), respectively, whereas a reduction in the incidence of renal failure of 30% resulted in an 8.4% decrease in overall cost (~€363 K).

Finally, the analyses on the parametric extrapolation of the overall survival curve and the renal failure incidence curve led to an increase of 0.3% (~€397 K) and 4.0% (~€411 K), respectively. This limited variation is due to the fact that the new extrapolations mostly impact the tails of the curves, where patients incur in low costs as they progressively decease and exit the model.

## Discussion

HBV is a major risk factor for liver cirrhosis and hepatocellular carcinoma in Italy (3), and liver transplantation (LT) is the best therapeutic option for patients with end-stage liver disease caused by HBV infection (64). Even though other studies have estimated the economic burden associated to LT due to different etiologies (65) and in different geographies (66), to the best of our knowledge, no previous analysis has investigated the lifetime cost for adult patients receiving a liver graft for HBV recipients in Italy or Europe. The modeling methodology we adopted followed a robust multi-step approach (9) from problem conceptualization to pragmatic literature review, model implementation, and model validation with a panel of national experts working in different Italian regions. Additionally, the model adopts a microsimulation approach to simulate the complex clinical pathway of this category of patients and provides statistically stable (15, 67) estimates of the average cost for patients undergoing LT in Italy in 2022.

The current analysis shows that the economic burden of LT for HBV-related diseases is high, with a discounted average lifetime cost per transplant patient of €395,986 and an overall impact for the Italian NHS of €92.3 million for the entire population of HBV patients transplanted in 2022. The greatest cost driver is end-stage renal failure, which accounts for 31.9% (~€126 k) of the total cost. Although this comorbidity has a 10-year cumulative incidence of less than 25% (61), as it also emerges from model results (Table 8), its management cost is high (~€33 k per year on average) (49–51) when considering dialysis and referral to renal transplantation (61). This evidence highlights the need for reinforcing “renal sparing” policies in the management of post liver transplant recipients in order to reduce both the incidence and the severity of renal complications, thus greatly impacting on total costs. The major cost drivers of liver transplantation are immunosuppression (20.6% of the total cost, ~€81 k) and liver transplant procedures (15.8% of the total cost, ~€63 k). HBV recurrence prophylaxis with HBIG and antiviral drugs accounts for 12.4 and 6.4% of the total cost, respectively, whereas all other cost components are residual. Additionally, sensitivity analyses have shown that considering the weighted average price of drugs instead of official drug prices leads to a reduction in total costs of 12.6% (~€346 k), with the relative contribution of anti-HBV recurrence prophylaxis of 13.2% instead of 18.8%. Furthermore, the lifetime duration of HBIG prophylaxis results in a limited cost increase (6.6%) versus −11.1% for the 1-year prophylaxis regimen, highlighting the maximum variation of costs resulting from the two limit durations adopted for patients with detectable HBV at time of transplantation in Italian clinical practice (36).

The reliability of the model was confirmed by sensitivity analyses, with variations ranging from €346,263 to €421,969 compared to the baseline value of €395,986/patient. Discount rate variations resulted in fluctuations between −17.9% (~€325 k) and + 47.0% (~€582 k) at 5 and 0% discount rates, respectively. The significant impact of discount rate variations is due to the long-term economic burden of LT, whereby consequences are spread over time and are consequently affected by higher discount rates.

While there are no published studies quantifying the overall lifetime cost for HBV patients undergoing LT in Italy, our analysis is consistent with previous Italian (68) and international (34, 69, 70) estimates of the cost components. To allow comparisons and cross-validation with these studies, costs reported in the literature were inflated to 2022 and adjusted for purchase power parity (PPP). Filipponi et al. (68) estimated the cost of transplantation and post-transplantation hospital stay during the acute phase. Considering a one-month acute-phase period, the corresponding cost from our model was aligned with this estimate (~€67 k vs. ~€77 k). Van der Hilst et al. and Longworth et al. (69, 70) estimated the LT-associated cost for a two-year follow-up at ~€122 k and ~ €95 k, respectively (versus ~€113 k in our analysis). Harries et al. (34) reported on the cost of waiting lists versus LT plus a three-year follow-up. Both costs were consistent with the results of our model, with estimates of ~€8 k and ~ €138 k, respectively, versus ~€15 k and ~ €126 k in the present study. Finally, Bjørnelv and co-authors (65) estimate the cost of liver transplantation for patients with colorectal metastases in Norway. When considering inflation and PPP, their lifetime cost estimation amounts to ~€180 k for the entire cohort of patients and ~ €200 k for a selected cohort, being largely lower than this study estimates. The difference may be explained by the shorter time horizon considered (25 years instead of lifetime), higher a discount rate (4.0% instead of 3.0%), the inclusion in their analysis of a lower number of cost components (transplantation and re-transplantation, post-operative complications, follow-up, immunosuppressive drugs, and anti-tumoral drugs in case of cancer recurrence), and clinical differences associated to the health system (Norway vs. Italian) and the etiology (colorectal metastases instead of HBV).

In addition to the validation by Italian KOLs in various phases of the study and the comparison with results from published analyses on costs related to liver transplantation, the model has been subject to other validations by the authors. Published data from observational studies and reports on LT in Italy allow for verification of model results in terms of incidence of comorbidities and complications and patient survival. Furthermore, the probabilities of occurrence reported in Table 8 are reflective of the incidence curves and survival curves extrapolated for model calibration (Tables 2, 3), highlighting the internal validity of the model ad well as the solidity of the microsimulation approach.

Despite its novelty and these validations, the model presents some limitations. First, some costs may have been underestimated. The model was constructed to estimate the average lifetime direct healthcare costs for HBV patients without any comorbidity or co-infection at the time of transplantation, thus considering a conservative resource-consumption scenario (32, 35). In addition, we assumed a maximum of one re-LT, as further re-transplantation is extremely rare (27). Finally, some estimates were based on the national tariffs in force in 2022, which have not been updated since 2013. This may lead to the underestimation of real costs (45, 71); however, it is the standard methodology for cost studies (72). To address this limitation, three sensitivity analyses were conducted varying the values of different cost components, which resulted in limited variations in total cost with respect to baseline.

In terms of model conceptualization and structure, there are some limitations due to the complexity of the LT clinical pathway of LT (15). Some post-transplant complications such as biliary complications and chronic rejection were not included in the model. Biliary complications encompass a wide spectrum of clinically variable scenarios (i.e., casts, stones, stenosis, leaks, and ischemic cholangiopathy) and cannot be easily encapsulated in a general model structure, while the incidence of chronic rejection is considered negligible in the modern era of immunosuppression regimens (4, 7, 21). Furthermore, the probabilities of complications were assumed to be independent of patient and donor characteristics, drug dosage regimen, and institutional framework and were independent of each other. This limitation in the model structure is due to the lack of highly detailed inputs and might lead to an overestimation of the cost of complications, as they are added on top of each other without considering that resource consumption increases less than proportionally with the increase in the number of complications (73).

Finally, the model was based on data from national guidelines and the literature, and although it had been validated with a panel of Italian experts, real-world evidence for further validation and verification of results is scarce.

In conclusion, this is the first study to estimate the lifetime direct healthcare cost of HBV patients undergoing LT in Italy and to provide data on the economic burden of liver transplantation for the Italian NHS. We firmly believe that the current results may pave the way for further research on this topic at a national level. The current model may be integrated with health technology assessment of new technologies introduced in transplantation, which may impact the cost trajectory of transplant patients. Furthermore, it may be adapted and adjusted to estimate costs related to LT for other indications (e.g., alcohol-associated liver disease and fatty liver disease). Finally, the analysis may be enriched by considering broader perspectives (including costs incurred by society and patients), with the involvement of patients and stakeholders.

## Data availability statement

The original contributions presented in the study are included in the article/Supplementary material, further inquiries can be directed to the corresponding author/s.

## Author contributions

AM: Writing – review & editing, Validation, Supervision, Conceptualization. BC: Methodology, Formal analysis, Writing – review & editing, Writing – original draft, Data curation, Conceptualization. LDC: Writing – review & editing, Validation, Supervision. PDS: Writing – review & editing, Validation, Supervision. FF: Methodology, Formal analysis, Writing – review & editing, Writing – original draft, Data curation, Conceptualization. MR: Writing – review & editing, Validation, Supervision. CV: Methodology, Formal analysis, Writing – review & editing, Writing – original draft, Data curation, Conceptualization. SF: Writing – review & editing, Validation, Supervision, Conceptualization.

## Footnotes

^1^Since at the time of the pragmatic literature review, MACEs had yet to be included in the patient flow, incidence curve for this comorbidity was retrieved in a second place through research of published literature and later validated with two KOLs.

^2^The model assumes that re-transplantation can only occur once during the patients’ lifetime.

^3^Calculated as the number of registered LT in Italy in 2022 [1,474 from the National Transplant Network report 2023 (19)] multiplied by the percentage of CHBV-related transplant from Italian literature [15.8% from Brancaccio et al., 2020 (18)].

^4^IQVIA statistical elaboration on consumptions and expenditure data from a panel of hospital pharmacies across the Italian territory.
