# Supplementary material for: Estimation of lifetime costs for patients receiving a transplant: the case of liver transplantation related to hepatitis B in Italy
Source: Front Public Health. 2024 Jul 3;12:1328782. doi: 10.3389/fpubh.2024.1328782 (PMC11256195; doi:10.3389/fpubh.2024.1328782)
Supplement: Supplementary file 1 [file Table_1.docx]

# **Supplementary material**

## **Pragmatic literature review**

Supplementary Material 1 illustrates the PRISMA flow diagram depicting the articles selection process. The initial research identified a total of 6,894 abstracts. Once excluded overlapping titles, 2,605 unique results remained. One reviewer screened the titles, eliminating 2,512 article which did not directly relate to the research question, were not published in English, or pertained to non-European countries. A second reviewer read the remaining abstracts to eliminate unsuitable publications not detected by title screening and 15 articles were eliminated, due to lack of full text availability. Finally, the second reviewer and a third reviewer read the full text articles (n=78) and categorized them in a template built in Microsoft Excel, extracting all relevant information.

Extracted information included: general information about the study (year of publication, first author, title, and study objective), a categorization of reported relevant inputs which could be used to populate the model, characteristics of the population studied in the paper (age, etiology for LT, subpopulations, etc.), and other relevant information about the study design (geographical setting, type of study, data sources used, number of patients/ transplantations, years of analysis).

**Supplementary Material 1. PRISMA flow diagram of pragmatic literature review**


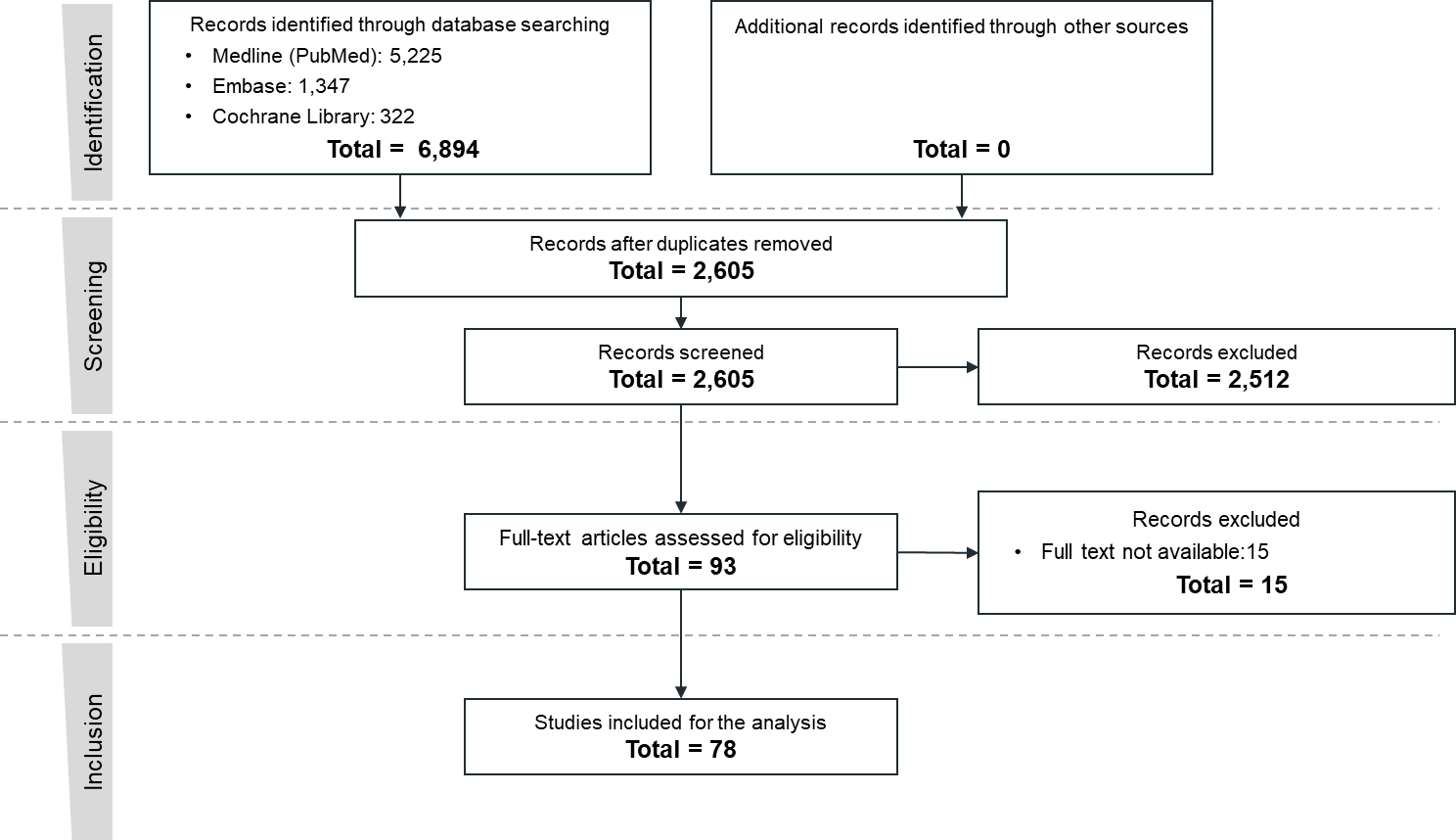


**Supplementary Material 2. Research strings on relevant inputs**

| **No.** | **String Definition** |
| --- | --- |
| **0^a^** | ((("Liver Transplantation"[Mesh]) OR ("liver transplantation"[Title/Abstract] OR "liver transplant*"[Title/Abstract] OR "Liver Grafting"[Title/Abstract] OR "Liver Transplantations"[Title/Abstract] OR "Liver Transplant"[Title/Abstract] OR "Liver Transplants"[Title/Abstract] OR "Hepatic Transplantation"[Title/Abstract] OR "Hepatic Transplant*"[Title/Abstract] OR "Hepatic Transplantations"[Title/Abstract])) AND (("Europe"[Mesh]) OR (((("Italy"[Mesh]) OR (Italy[Title/Abstract])) OR (Italia[Title/Abstract])) OR (Itali*[Title/Abstract]))) |
| **1** | (((((("Waiting Lists"[Mesh]) OR ("Waiting List"[Title/Abstract] OR "Waiting Lists"[Title/Abstract]))) OR ((wait*[Title/Abstract]) AND ((list*[Title/Abstract] OR time[Title/Abstract] OR period)[Title/Abstract]))) OR (waitlist*[Title/Abstract])) OR ((("Postoperative Period"[Mesh]) OR "Preoperative Period"[Mesh]) OR (((pre[Title/Abstract] OR post[Title/Abstract] OR after[Title/Abstract] OR before)[Title/Abstract]) AND ((operat*[Title/Abstract] OR surgery[Title/Abstract] OR transplant*)[Title/Abstract]))))) |
| **2** | ((((("Postoperative Complications"[Mesh]) OR ("Graft Rejection"[Mesh])) OR ("Postoperative Complication*"[Title/Abstract] OR "Graft Rejection*"[Title/Abstract]) OR (((operat*[Title/Abstract] OR surgery[Title/Abstract] OR transplant*[Title/Abstract] OR graft*)[Title/Abstract]) AND ((Rejection*[Title/Abstract] OR complication*[Title/Abstract] OR failure[Title/Abstract] OR success[Title/Abstract] OR infection*[Title/Abstract] OR Malignancy[Title/Abstract] OR Recurrence)[Title/Abstract] OR “re-transplantation”[Title/Abstract] OR "second liver transplant"[Title/Abstract] OR retransplantation[Title/Abstract])))) |
| **3** | (((("Recurrence"[Mesh]) OR ("Neoplasm Metastasis"[Mesh:NoExp])) OR ("Mortality"[Mesh])) OR ((((operat*[Title/Abstract] OR surgery[Title/Abstract] OR transplant*[Title/Abstract] OR graft*)[Title/Abstract])) AND ("Chronic Liver Failure*"[Title/Abstract] OR Metastas*[Title/Abstract] AND recurren*[Title/Abstract] OR Recrudescence*[Title/Abstract] OR Relapse*[Title/Abstract] OR Reinfection*[Title/Abstract] OR "Re-infection*" [Title/Abstract] OR "Re infection*" [Title/Abstract] OR "Recurrent Infection*" [Title/Abstract] OR novo[Title/Abstract])))) |
| **4** | (("Costs and Cost Analysis"[Mesh] OR "Cost-Benefit Analysis"[Mesh] OR "Economics, Medical"[Mesh]) OR (("Cost-Benefit Analyses"[Title/Abstract] OR "Cost Benefit Analysis"[Title/Abstract] OR "Cost Benefit Analyses"[Title/Abstract] OR "Cost Effectiveness"[Title/Abstract] OR "Cost-Benefit Data"[Title/Abstract] OR "Cost Benefit Data"[Title/Abstract] OR "Cost-Utility Analysis"[Title/Abstract] OR "Cost Utility Analysis"[Title/Abstract] OR "Cost-Utility Analyses"[Title/Abstract] OR "Economic Evaluation"[Title/Abstract] OR "Economic Evaluations"[Title/Abstract] OR "Marginal Analysis"[Title/Abstract] OR "Marginal Analyses"[Title/Abstract] OR "Cost Benefit"[Title/Abstract] OR "Costs[Title/Abstract] OR Benefits"[Title/Abstract] OR "Benefits[Title/Abstract] AND Costs"[Title/Abstract] OR "Cost-Effectiveness Analysis"[Title/Abstract] OR "Cost Effectiveness Analysis"[Title/Abstract] OR "Costs[Title/Abstract] AND Cost Analyses"[Title/Abstract] OR "Cost Analysis"[Title/Abstract] OR "Cost Analyses"[Title/Abstract] OR "Cost Comparison"[Title/Abstract] OR "Cost Comparisons"[Title/Abstract] OR Affordability[Title/Abstract] OR Affordabilities[Title/Abstract] OR "Cost-Minimization Analysis"[Title/Abstract] OR "Cost Minimization Analysis"[Title/Abstract] OR "Cost-Minimization Analyses"[Title/Abstract] OR Pricing[Title/Abstract] OR Cost[Title/Abstract] OR Costs[Title/Abstract] OR "Cost Measures"[Title/Abstract] OR "Cost Measure"[Title/Abstract] OR "Decision analysis"[Title/Abstract] OR "cost-effectiveness"[Title/Abstract] OR "Cost-Effectiveness"[Title/Abstract] OR "Clinical Effectiveness"[Title/Abstract]))) |
| **5** | (("Follow-Up Studies"[Mesh]) OR ((((("Graft Survival"[Mesh]) OR ("Life Style"[Mesh:NoExp])) OR ("Graft Survival*"[Title/Abstract])) OR ((patient*[Title/Abstract]) AND ((management [Title/Abstract] OR "Life Style*"[Title/Abstract] OR Lifestyle*)[Title/Abstract])))))) |
| Notes: a) String 0 was searched both on its own and in combination with strings 1-5. Inputs already found with string 0 were found in each combination, with a higher level of detail pertaining to the objective of the string. | |

**Supplementary Material 3. Research strings on relevant study types**

| **Type of study** | **String Definition** |
| --- | --- |
| **SR&MA and RCT** | ((((((((("Meta-Analysis as Topic"[Mesh]) OR "Meta-Analysis"[Publication Type]) OR "meta-analysis[Title] OR systematic review"[Title] OR "meta analy*"[Title] OR "metaanaly*"[Title] OR "Meta-Analysis"[Title])) OR ("systematic literature review"[Title])) OR ("systematic review"[Title/Abstract])) OR (((("Randomized Controlled Trial"[Publication Type]) OR "Randomized Controlled Trials as Topic"[Mesh]) OR ("Clinical Trial" [Publication Type])) OR (randomized[Title/Abstract] OR randomised[Title/Abstract] OR randomly[Title/Abstract]))))) |
| **Prospective and Retrospective** | ((("Cohort Studies"[Mesh]) OR "Epidemiologic Studies"[Mesh]) OR ((Cohort [Title/Abstract] OR observational[Title/Abstract] OR prospective[Title/Abstract] OR retrospective[Title/Abstract]) AND (study[Title/Abstract] OR studies[Title/Abstract])))) NOT "case control"[Title] |
| Acronyms: SR&MA = Systematic Review and Meta-Analysis; RCT = Randomized Controlled Trial | |

**Supplementary Material 4. Sources identified through pragmatic literature review, by cost component**

|  | **1^st^ best** | **2^nd^ best** | **3^rd^ best** |
| --- | --- | --- | --- |
| ***Waiting list*** | - Khouzam et al., 2019 (1) - Angelico et al., 2019 (2) - Harries et al., 2019 (3) | - Harries et al., 2017 (4) - Gyori et al., 2016 (5) | - Fosby et al., 2015 (6) - Finkenstedt et al., 2013 (7) - Rauchfuss et al., 2013 (8) - Cadahia et al., 2010 (9) |
| ***Patients’ Survival*** | - Takagi et al., 2020 (10) - Wallace et al., 2020 (11) - Haldar et al., 2019 (12) - Carbone et al., 2016 (13) - Vitale et al., 2015 (14) | - Adam et al., 2019 (15) - Cillo et al., 2019 (16) - Colombo et al., 2019 (17) - Adam et al., 2018 (18) - Gagliotti et al., 2018 (19) - Czerwinski et al., 2018 (20) - Levesque et al., 2017 (21) - Gyori et al., 2016 (5) - Memeo et al., 2016 (22) - Schoening et al., 2016 (23) - Tinoco-González et al., 2016 (24) - Aberg et al., 2015 (25) - Antunes et al., 2015 (26) - Bosch et al., 2015 (27) - Fischer et al., 2015 (28) - Fosby et al, 2015 (6) - Farinati et al., 2012 (29) - Ettorre et al., 2011 (30) - Baccarini et al., 2010 (31) - Pauwelyn et al., 2010 (32) | - Briggs et al., 2021 (33) - Belli et al., 2018 (34) - Kulik et al., 2017 (35) - Rostved et al., 2016 (36) - Finkenstedt et al., 2013 (7) - Rauchfuss et al., 2013 (8) - Saliba et al., 2013 (37) - Germani et al., 2012 (38) - Marín-Gómez et al., 2012 (39) - Suzuki et al., 2012 (40) - Aberg et al., 2011 (41) - Aberg et al., 2010 (42) - Baccarani et al., 2010 (31) - Bert et al., 2010 (43) - Cadahìa et al., 2010 (9) - Sersté et al., 2010 (44) |
| ***Acute infections*** | - Freire et al., 2021 (45) - Gagliotti et al., 2018 (19) | - Kusejko et al., 2021 (46) - Bardou et al., 2019 (47) - Brustia et al., 2019 (48) - Adam et al., 2018 (18) - Angelico et al., 2018 (49) - Busch et al., 2018 (50) - Carbone et al., 2016 (13) - Aberg et al., 2015 (25) - Ambrus et al., 2015 (51) - Antunes et al., 2015 (26) - Ettorre et al., 2011 (30) | - Fischer et al., 2015 (28) - Bodro et al., 2012 (52) - Germani et al., 2012 (38) - Aberg et al., 2011 (41) - Boudjema et al., 2011 (53) - Aberg et al., 2010 (42) - Bert et al., 2010 (43) |
| ***Liver rejection*** | - Haldar et al., 2019 (12) - Carbone et al., 2016 (13) - Rostved et al., 2016 (36) - Tinoco-González et al., 2016 (24) | - Briggs et al., 2021 (33) - Wallace et al., 2020 (11) - Adam et al., 2019 (15) - Angelico et al., 2019 (2) - Cillo et al., 2019 (16) - Adam et al., 2018 (18) - Czerwinski et al., 2018 (20) - Schoening et al., 2016 (23) - Bosch et al., 2015 (27) - Baccarani et al., 2010 (54) | - Kulik et al., 2017 (35) - Levesque et al., 2017 (21) - Belli et al., 2015 (34) - Germani et al., 2012 (38) - Boudjema et al., 2011 (53) - Aberg et al., 2010 (42) |
| ***Re-transplantation*** | - Takagi et al., 2020 (10) | - Kusejko et al., 2021 (46) - Adam et al., 2018 (18) - Kulik et al., 2017 (35) - Memeo et al., 2016 (22) | - Harries et al., 2019 (3) - Belli et al., 2018 (34) |
| ***HBV recurrence*** | - Pauwelyn et al., 2010 (32) | - Bosh et al., 2015 (27) | - Bardou et al., 2019 (47) - Brustia et al., 2019 (48) - Adam et al., 2018 (18) - Angelico et al., 2018 (49) - Carbone et al., 2016 (13) - Aberg et al., 2015 (25) - Ambrus et al., 2015 (51) - Ettorre et al., 2011 (30) |
| **De novo *malignancies*** | - Taborelli et al., 2018 (55) - Baccarani et al., 2010 (31) | - Schoening et al., 2016 (23) - Tovikkai et al., 2015 (56) | - Bardou et al., 2019 (47) - Brustia et al., 2019 (48) - Adam et al., 2018 (18) - Angelico et al., 2018 (49) - Carbone et al., 2016 (13) - Fisher et al., 2015 (28) - Aberg et al., 2015 (25) - Ambrus et al., 2015 (51) - Ettorre et al., 2013 (30) - Germani et al., 2012 (38) - Boudjema et al., 2011 (53) - Ettorre et al., 2011 (30) - Aberg et al., 2010 (42) |
| ***Renal failure*** | - Boudjema et al., 2011 (53) | - Colombo et al., 2019 (17) - Fischer et al., 2015 (28) | - Bardou et al., 2019 (47) - Brustia et al., 2019 (48) - Adam et al., 2018 (18) - Angelico et al., 2018 (49) - Carbone et al., 2016 (13) - Aberg et al., 2015 (25) - Ambrus et al., 2015 (51) - Saliba et al., 2013 (37) - Germani et al., 2012 (38) - Ettorre et al., 2011 (30) - Aberg et al., 2010 (42) |
| ***Diabetes*** | - Boudjema et al., 2011 (53) | - | - Bardou et al., 2019 (47) - Brustia et al., 2019 (48) - Adam et al., 2018 (18) - Angelico et al., 2018 (49) - Carbone et al., 2016 (13) - Fisher et al., 2015 (28) - Aberg et al., 2015 (25) - Ambrus et al., 2015 (51) - Germani et al., 2012 (38) - Ettorre et al., 2011 (30) - Aberg et al., 2010 (42) |
| First best choices were considered papers containing relevant information according to our scope, published from 2015 onwards, with data on HBV patients without co-infections, with a focus on Italy, and with a significant sample of patients (≥ 50 patients). Second best choices were considered papers containing relevant information, but more dated or less specific than the ones already identified as “first best-choice”, or records reporting less relevant information than first best choice records, but more specific (e.g., focus on Italy). Finally, third best choices were considered publications not containing relevant information pertaining to our scope, old records, or records with data on co-infected patients. | | | |

## **Sample size and number of iterations justification**

**Supplementary Material 5. Standard error if average total cost resulting from different number of iterations, by cohort size**


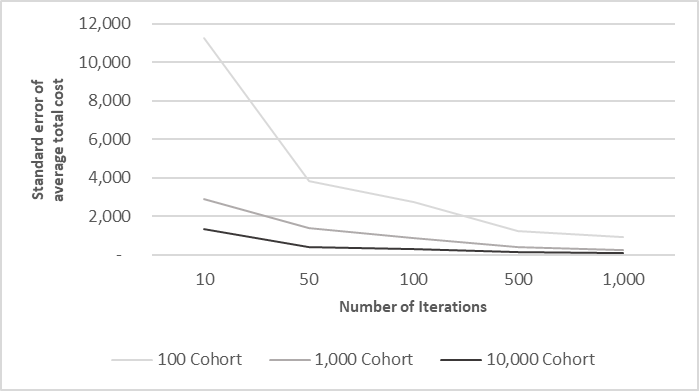


## **Parametric models for incidence and survival curves**

**Supplementary Material 6. AIC and BIC of parametric models analyzed for the model's incidence curves**

| **Parametric Model** | **AIC** | **BIC** | |
| --- | --- | --- | --- |
| **Liver Rejection** (12) |  |  | |
| *Exponential* | 13279.74 | 13286.15 | |
| *Gamma* | 11885.22 | 11898.03 | |
| *Gompertz* | 12538.72 | 12551.53 | |
| *Log-Logistic* | 11835.47 | 11848.29 | |
| *Log-Normal* | 11733.41 | 11746.22 | |
| *Weibull* | 11858.81 | 11871.62 | |
| *Hazard 2 knots* | 11305.66 | 11331.29 | |
| ***Hazard 1 knots*** | **11770.68** | **11789.89** | |
| **HBV Recurrence** (32) |  |  | |
| *Exponential* | 97.53 | 99.56 | |
| *Gamma* | 99.42 | 103.47 | |
| ***Gompertz*** | **99.44** | **103.49** | |
| *Log-Logistic* | 99.41 | 103.46 | |
| *Log-Normal* | 99.19 | 103.24 | |
| *Weibull* | 99.41 | 103.47 | |
| *Hazard 2 knots* | 102.91 | 111.01 | |
| *Hazard 1 knots* | 101.28 | 107.35 | |
| ***De novo* malignancies** (55) |  |  | |
| *Exponential* | 2662.39 | 2668.33 | |
| *Gamma* | 2657.08 | 2668.98 | |
| ***Gompertz*** | **2647.43** | **2659.33** | |
| *Log-Logistic* | 2655.44 | 2667.33 | |
| *Log-Normal* | 2646.32 | 2658.22 | |
| *Weibull* | 2656.60 | 2668.50 | |
| *Hazard 2 knots* | 2647.80 | 2671.59 | |
| *Hazard 1 knots* | 2646.03 | 2663.87 | |
| **Renal failure** (57) |  |  | |
| *Exponential* | 141696.45 | 141704.96 | |
| *Gamma* | 140991.42 | 141008.45 | |
| ***Gompertz*** | **140809.06** | **140826.09** | |
| *Log-Logistic* | 140799.38 | 140816.41 | |
| *Log-Normal* | 140291.85 | 140308.88 | |
| *Weibull* | 140925.39 | 140942.42 | |
| *Hazard 2 knots* | 138964.03 | 138998.08 | |
| *Hazard 1 knots* | 140193.50 | 140219.05 | |
| **Diabetes** (53) **^a^** |  |  | |
| *Exponential* | 512.46 | 515.04 | |
| *Gamma* | 470.46 | 475.62 | |
| ***Gompertz*** | **452.31** | **457.47** | |
| *Log-Logistic* | 465.66 | 470.81 | |
| *Log-Normal* | 462.08 | 467.24 | |
| *Weibull* | 468.14 | 473.30 | |
| *Hazard 2 knots* | 452.44 | 462.76 | |
| *Hazard 1 knots* | 453.01 | 460.74 | |
| **MACEs** (58) |  |  | |
| *Exponential* | 1383.49 | 1388.14 | |
| *Gamma* | 1332.72 | 1342.02 | |
| ***Gompertz*** | **1298.52** | **1307.82** | |
| *Log-Logistic* | 1327.36 | 1336.66 | |
| *Log-Normal* | 1317.88 | 1327.19 | |
| *Weibull* | 1330.33 | 1339.64 | |
| *Hazard 2 knots* | 1307.34 | 1325.96 | |
| *Hazard 1 knots* | 1311.32 | 1325.28 | |
| Acronyms: MACEs = Major Adverse Cardiovascular Events  Notes: a) Weighted average between experimental and control group incidence curves from the study of interest.  The selected parametric model for each curve is highlighted in bold. | | |  |

**Supplementary Material 7. Graphic representation of parametric models analyzed for the model's incidence curves**

**
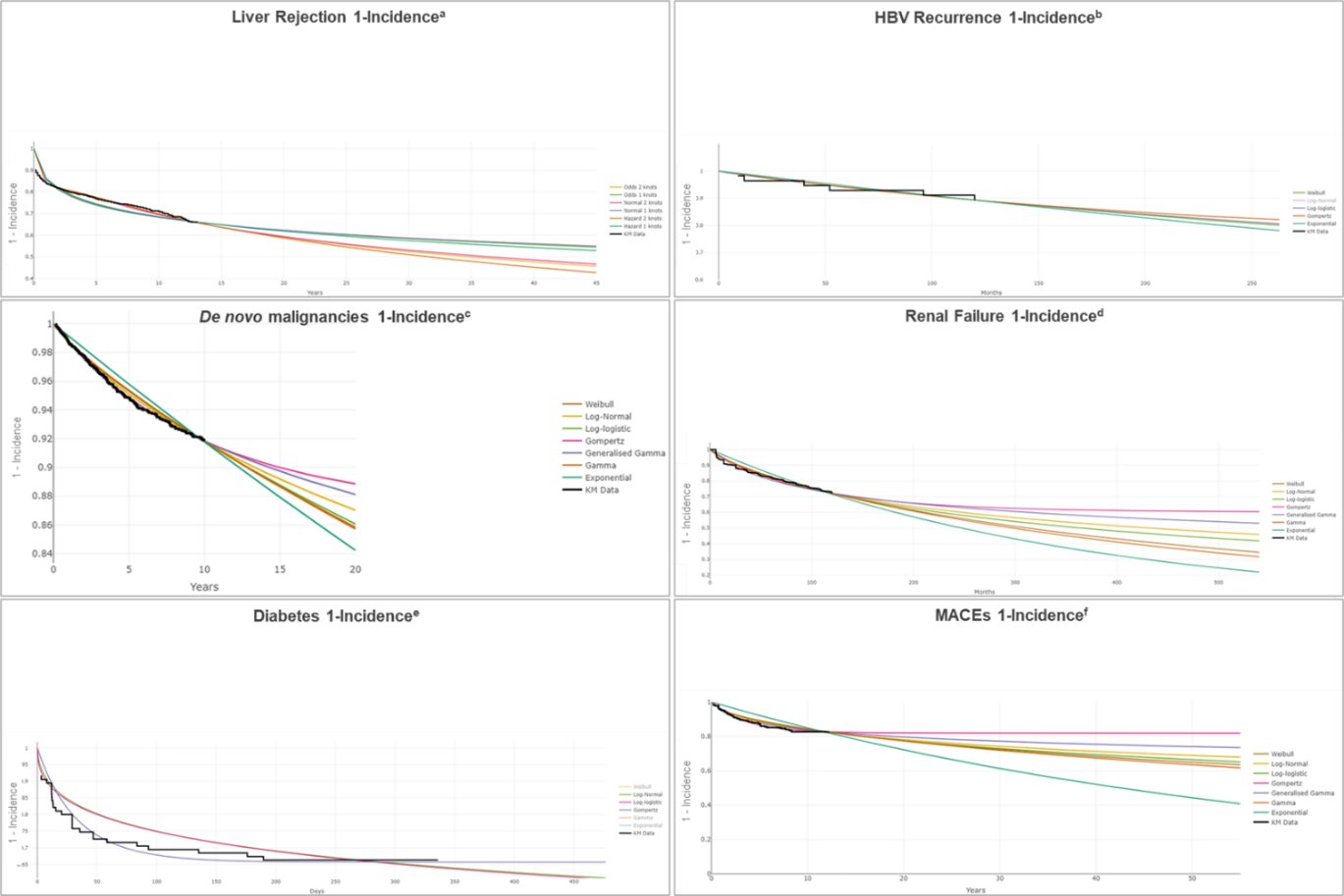
**

Sources: a) Haldar et al., 2019 (12); b) Pauwelyn et al., 2010 (32); c) Taborelli et al, 2018 (55); d) Ojo et al., 2003 (57); e) Boudjema et al., 2011 (53) – only curve for experimental arm in the study is shown; f) Albedawi et al., 2012 (58)

Notes: Selected parametric models a) Hazard 1 knot; b) Gompertz; c) Gompertz; d) Gompertz; e) Gompertz; f) Gompertz

**Supplementary Material 8. AIC and BIC of parametric models analyzed for the model's survival curves**

| **Parametric Model** | **AIC** | **BIC** |
| --- | --- | --- |
| **Overall Survival** (12) |  |  |
| *Exponential* | 12198.92 | 12205.33 |
| *Gamma* | 11229.05 | 11241.86 |
| *Gompertz* | 11717.82 | 11730.63 |
| *Log-Logistic* | 11206.30 | 11219.11 |
| *Log-Normal* | 11132.68 | 11145.49 |
| *Weibull* | 11216.07 | 11228.88 |
| ***Hazard 2 knots*** | **10773.12** | **10798.74** |
| *Hazard 1 knots* | 11175.89 | 11195.10 |
| **Re-transplantation** (10) |  |  |
| *Exponential* | 12379.49 | 12385.26 |
| *Gamma* | 11587.67 | 11599.23 |
| *Gompertz* | 12310.85 | 12322.41 |
| *Log-Logistic* | 11795.84 | 11807.40 |
| *Log-Normal* | 11790.46 | 11802.02 |
| *Weibull* | 11646.21 | 11657.76 |
| ***Hazard 2 knots*** | **11215.85** | **11238.96** |
| *Hazard 1 knots* | 11596.72 | 11614.06 |
| ***De novo* malignancies** (31) |  |  |
| *Exponential* | 2077.82 | 2081.85 |
| *Gamma* | 1947.41 | 1955.47 |
| *Gompertz* | 1859.29 | 1867.36 |
| *Log-Logistic* | 1915.91 | 1923.98 |
| *Log-Normal* | 1901.66 | 1909.73 |
| *Weibull* | 1933.63 | 1941.70 |
| ***Hazard 2 knots*** | **1862.92** | **1879.05** |
| *Hazard 1 knots* | 1874.95 | 1887.05 |
| **MACEs** (56) |  |  |
| *Exponential* | 15586.49 | 15592.75 |
| *Gamma* | 15092.58 | 15105.08 |
| *Gompertz* | 15566.99 | 15579.50 |
| *Log-Logistic* | 15362.98 | 15375.48 |
| *Log-Normal* | 15453.87 | 15466.38 |
| *Weibull* | 15153.46 | 15165.96 |
| ***Hazard 2 knots*** | **14600.37** | **14625.38** |
| *Hazard 1 knots* | 15084.07 | 15102.83 |
| Note: The selected parametric model for each curve is highlighted in bold | | |

**Supplementary Material 9. Graphic representation of parametric models analyzed for the model's survival curves**


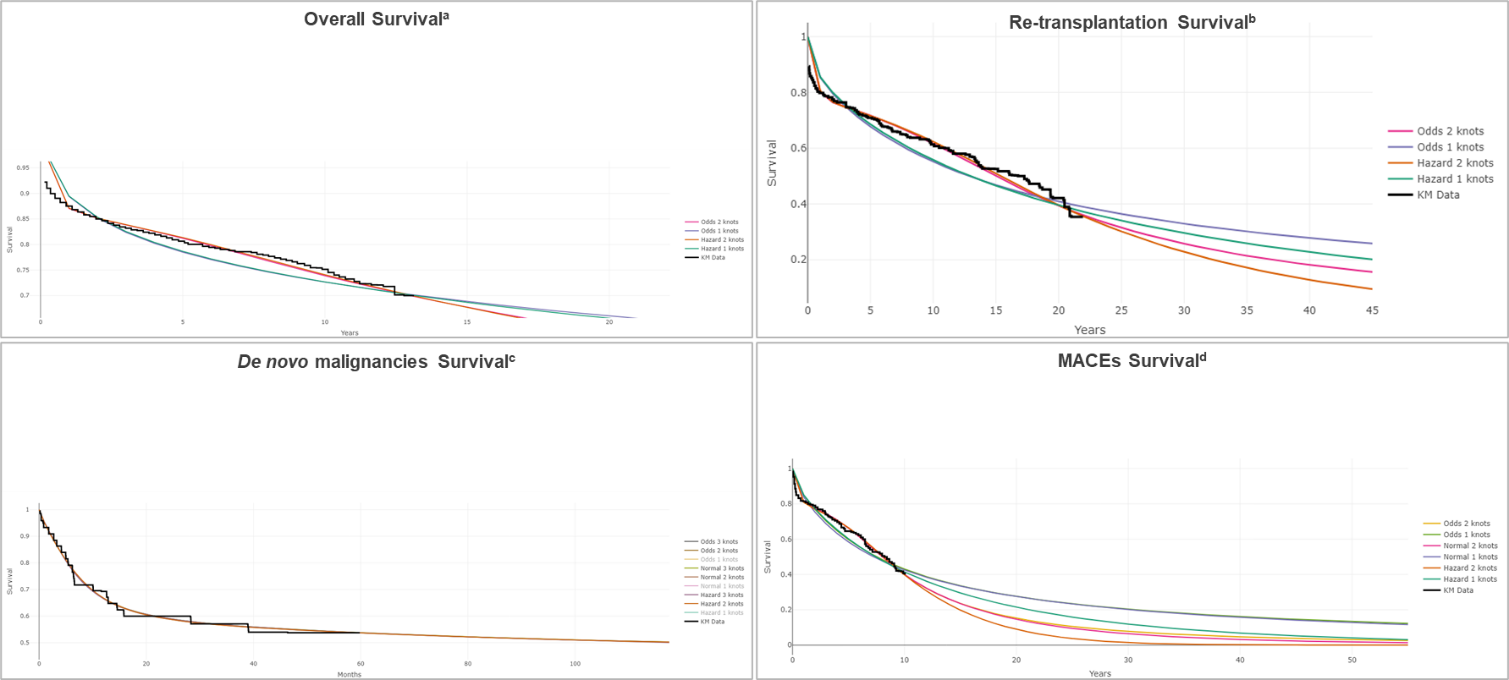


Sources: a) Haldar et al., 2019 (12); b) Tagaki et al., 2020 (10); c) Baccarani et al, 2010 (31); d) Tovikkai et al., 2015 (56)

Notes: Selected parametric models: a) Hazard 2 knots; b) Hazard 2 knots; c) Hazard 2 knots; d) Hazard 2 knots

# **References**

1. Khouzam S, Pagano D, Barbàra M, Cintorino D, Li Petri S, Di Francesco F, et al. Impact of Italian Score for Organ Allocation System on Deceased Donor Liver Transplantation: A Monocentric Competing Risk Time-to-Event Analysis. Transplantation Proceedings. 2019 Nov;51(9):2860–4.

2. Angelico R, Trapani S, Spada M, Colledan M, De Ville De Goyet J, Salizzoni M, et al. A national mandatory-split liver policy: A report from the Italian experience. American Journal of Transplantation. 2019 Jul;19(7):2029–43.

3. Harries L, Gwiasda J, Qu Z, Schrem H, Krauth C, Amelung VE. Potential savings in the treatment pathway of liver transplantation: an inter-sectorial analysis of cost-rising factors. Eur J Health Econ. 2019 Mar;20(2):281–301.

4. Harries L, Schrem H, Stahmeyer JT, Krauth C, Amelung VE. High resource utilization in liver transplantation-how strongly differ costs between the care sectors and what are the main cost drivers?: a retrospective study. Transpl Int. 2017 Jun;30(6):621–37.

5. Györi GP, Silberhumer GR, Rahmel A, De Vries E, Soliman T, Zehetmayer S, et al. Impact of dynamic changes in MELD score on survival after liver transplantation - a Eurotransplant registry analysis. Liver Int. 2016 Jul;36(7):1011–7.

6. Fosby B, Melum E, Bjøro K, Bennet W, Rasmussen A, Andersen IM, et al. Liver transplantation in the Nordic countries – An intention to treat and post-transplant analysis from The Nordic Liver Transplant Registry 1982–2013. Scandinavian Journal of Gastroenterology. 2015 Jun 3;50(6):797–808.

7. Finkenstedt, Nachbaur, Zoller, Joannidis, Pratschke, Graziadei, et al. Acute-on-Chronic Liver Failure: Excellent Outcomes After Liver Transplantation but High Mortality on the Wait List. 2013;

8. Rauchfuss, Zidan, Scheuerlein, Dittmar, Bauschke, Settmacher. Waiting time, not donor-risk-index, is a major determinant for beneficial outcome after liver transplantation in high-MELD patients. Ann Transplant. 2013;18:243–7.

9. Cadahía V, González-Diéguez ML, Alonso P, García-Bernardo C, Miyar De León A, Barneo L, et al. Exclusions and Deaths on the Liver Transplant Waiting List. Transplantation Proceedings. 2010 Mar;42(2):622–4.

10. Takagi K, Domagala P, Porte RJ, Alwayn I, Metselaar HJ, Van Den Berg AP, et al. Liver retransplantation in adult recipients: analysis of a 38‐year experience in the Netherlands. J Hepato Biliary Pancreat. 2020 Jan;27(1):26–33.

11. Wallace D, Cowling TE, Walker K, Suddle A, Rowe I, Callaghan C, et al. Short- and long-term mortality after liver transplantation in patients with and without hepatocellular carcinoma in the UK. British Journal of Surgery. 2020 May 28;107(7):896–905.

12. Haldar D, Kern B, Hodson J, Armstrong MJ, Adam R, Berlakovich G, et al. Outcomes of liver transplantation for non-alcoholic steatohepatitis: A European Liver Transplant Registry study. Journal of Hepatology. 2019 Aug;71(2):313–22.

13. Carbone M, Nardi A, Marianelli T, Martin K, Hudson A, Collett D, et al. International comparison of liver transplant programmes: differences in indications, donor and recipient selection and outcome between Italy and UK. Liver Int. 2016 Oct;36(10):1481–9.

14. Vitale, Spolverato, Burra, De Feo, Belli, Donato, et al. Cost-effectiveness of pretransplant sofosbuvir for preventing recurrent hepatitis C virus infection after liver transplantation. 2015 Apr 30;

15. Adam R, Karam V, Cailliez V, Trunečka P, Samuel D, Tisone G, et al. Improved Survival in Liver Transplant Patients Receiving Prolonged-release Tacrolimus-based Immunosuppression in the European Liver Transplant Registry (ELTR): An Extension Study. Transplantation. 2019 Sep;103(9):1844–62.

16. Cillo U, Saracino L, Vitale A, Bertacco A, Salizzoni M, Lupo F, et al. Very Early Introduction of Everolimus in De Novo Liver Transplantation: Results of a Multicenter, Prospective, Randomized Trial. Liver Transpl. 2019 Feb;25(2):242–51.

17. Colombo D, Zullo A, Simoni L, Zagni E. The SURF (Italian observational study for renal insufficiency evaluation in liver transplant recipients): a post-hoc between-sex analysis. BMC Nephrol. 2019 Dec;20(1):475.

18. Adam R, Karam V, Cailliez V, O Grady JG, Mirza D, Cherqui D, et al. 2018 Annual Report of the European Liver Transplant Registry (ELTR) - 50-year evolution of liver transplantation. Transpl Int. 2018 Dec;31(12):1293–317.

19. Gagliotti C, Morsillo F, Moro ML, Masiero L, Procaccio F, Vespasiano F, et al. Infections in liver and lung transplant recipients: a national prospective cohort. Eur J Clin Microbiol Infect Dis. 2018 Mar;37(3):399–407.

20. Czerwiński J, Lewandowska D, Małkowski P, Danielewicz R. Liver Transplant Registry in Poland: Web-Netted Quality Tool in Liver Transplantation From Deceased and Living Donors. Transplantation Proceedings. 2016 Jun;48(5):1347–9.

21. Levesque E, Winter A, Noorah Z, Daurès J, Landais P, Feray C, et al. Impact of acute‐on‐chronic liver failure on 90‐day mortality following a first liver transplantation. Liver International. 2017 May;37(5):684–93.

22. Memeo R, Laurenzi A, Pittau G, Sanchez-Cabus S, Vibert E, Adam R, et al. Repeat liver retransplantation: rationale and outcomes. Clin Transplant. 2016 Mar;30(3):312–9.

23. Schoening W, Helbig M, Buescher N, Andreou A, Schmitz V, Bahra M, et al. Eurotransplant donor‐risk‐index and recipient factors: influence on long‐term outcome after liver transplantation – A large single‐center experience. Clinical Transplantation. 2016 May;30(5):508–17.

24. Tinoco-González J, Suárez-Artacho G, Bernal-Bellido C, Cepeda-Franco C, Ramallo-Solis I, Marín-Gómez L, et al. Analysis of the First 1000 Liver Transplants in Virgen del Rocío Hospital. Transplantation Proceedings. 2016 Nov;48(9):2973–6.

25. Åberg F, Gissler M, Karlsen TH, Ericzon B, Foss A, Rasmussen A, et al. Differences in long‐term survival among liver transplant recipients and the general population: A population‐based nordic study. Hepatology. 2015 Feb;61(2):668–77.

26. Antunes M, Teixeira A, Fortuna P, Moya B, Martins A, Bagulho L, et al. Infections After Liver Transplantation: A Retrospective, Single-center Study. Transplantation Proceedings. 2015 May;47(4):1019–24.

27. Bosch A, Dumortier J, Maucort-Boulch D, Scoazec JY, Wendum D, Conti F, et al. Preventive administration of UDCA after liver transplantation for primary biliary cirrhosis is associated with a lower risk of disease recurrence. Journal of Hepatology. 2015 Dec;63(6):1449–58.

28. Fischer L, Saliba F, Kaiser GM, De Carlis L, Metselaar HJ, De Simone P, et al. Three-year Outcomes in De Novo Liver Transplant Patients Receiving Everolimus With Reduced Tacrolimus: Follow-Up Results From a Randomized, Multicenter Study. Transplantation. 2015 Jul;99(7):1455–62.

29. Farinati F, Giacomin A, Vanin V, Sergio A, Burra P, Cillo U, et al. Liver transplantation for hepatocellular carcinoma in clinical practice: the lesson from a 20-year multicentre experience in Italy. European Journal of Gastroenterology & Hepatology. 2012 Feb;24(2):195–202.

30. Ettorre GM, Santoro R, Vennarecci G, Lepiane P, Antonini M, Santoro E. Results of a newborn liver transplant program in the era of piggyback technique and extended donor criteria in Italy. Updates Surg. 2011 Sep;63(3):191–200.

31. Baccarani U, Piselli P, Serraino D, Adani GL, Lorenzin D, Gambato M, et al. Comparison of de novo tumours after liver transplantation with incidence rates from Italian cancer registries. Digestive and Liver Disease. 2010 Jan;42(1):55–60.

32. Pauwelyn K, Cassiman D, Laleman W, Verslype C, Monbaliu D, Aerts R, et al. Outcomes of Long-Term Administration of Intravenous Hepatitis B Immunoglobulins for the Prevention of Recurrent Hepatitis B After Liver Transplantation. Transplantation Proceedings. 2010 Dec;42(10):4399–402.

33. Briggs G, Wallace D, Flasche S, Walker K, Cowling T, Heaton N, et al. Inferior outcomes in young adults undergoing liver transplantation – a UK and Ireland cohort study. Transpl Int. 2021 Nov;34(11):2274–85.

34. Belli LS, Perricone G, Adam R, Cortesi PA, Strazzabosco M, Facchetti R, et al. Impact of DAAs on liver transplantation: Major effects on the evolution of indications and results. An ELITA study based on the ELTR registry. Journal of Hepatology. 2018 Oct;69(4):810–7.

35. Kulik U, Lehner F, Klempnauer J, Borlak J. Primary non‐function is frequently associated with fatty liver allografts and high mortality after re‐transplantation. Liver International. 2017 Aug;37(8):1219–28.

36. Rostved AA, Lundgren JD, Hillingsø J, Peters L, Mocroft A, Rasmussen A. MELD score measured day 10 after orthotopic liver transplantation predicts death and re-transplantation within the first year. Scandinavian Journal of Gastroenterology. 2016 Nov 1;51(11):1360–6.

37. Saliba F, De Simone P, Nevens F, De Carlis L, Metselaar HJ, Beckebaum S, et al. Renal Function at Two Years in Liver Transplant Patients Receiving Everolimus: Results of a Randomized, Multicenter Study. American Journal of Transplantation. 2013 Jul;13(7):1734–45.

38. Germani G, Theocharidou E, Adam R, Karam V, Wendon J, O’Grady J, et al. Liver transplantation for acute liver failure in Europe: Outcomes over 20years from the ELTR database. Journal of Hepatology. 2012 Aug;57(2):288–96.

39. Marín-Gómez LM, Bernal-Bellido C, Álamo-Martínez JM, Porras-López FM, Suárez-Artacho G, Serrano-Diaz-Canedo J, et al. Intraoperative Hepatic Artery Blood Flow Predicts Early Hepatic Artery Thrombosis After Liver Transplantation. Transplantation Proceedings. 2012 Sep;44(7):2078–81.

40. Suzuki H, Bartlett ASR, Muiesan P, Jassem W, Rela M, Heaton N. High Model for End-Stage Liver Disease Score as a Predictor of Survival During Long-Term Follow-up After Liver Transplantation. Transplantation Proceedings. 2012 Mar;44(2):384–8.

41. Åberg F, Mäklin S, Räsänen P, Roine RP, Sintonen H, Koivusalo AM, et al. Cost of a quality-adjusted life year in liver transplantation: The influence of the indication and the model for end-stage liver disease score: Cost Per QALY in Liver Transplantation. Liver Transpl. 2011 Nov;17(11):1333–43.

42. Åberg F, Jula A, Höckerstedt K, Isoniemi H. Cardiovascular Risk Profile of Patients With Acute Liver Failure After Liver Transplantation When Compared With the General Population. Transplantation. 2010 Jan 15;89(1):61–8.

43. Bert F, Larroque B, Paugam-Burtz C, Janny S, Durand F, Dondero F, et al. Microbial epidemiology and outcome of bloodstream infections in liver transplant recipients: An analysis of 259 episodes: Bloodstream Infection and Liver Transplantation. Liver Transpl. 2010 Mar;16(3):393–401.

44. Sersté T, Moreno C, Francoz C, Razek WA, Paugham C, Belghitti J, et al. The impact of preoperative hepatic hydrothorax on the outcome of adult liver transplantation. European Journal of Gastroenterology & Hepatology. 2010 Feb;22(2):207–12.

45. Freire MP, Song ATW, Oshiro ICV, Andraus W, D’Albuquerque LAC, Abdala E. Surgical site infection after liver transplantation in the era of multidrug-resistant bacteria: what new risks should be considered? Diagnostic Microbiology and Infectious Disease. 2021 Jan;99(1):115220.

46. Kusejko K, Neofytos D, Hirsch HH, Meylan P, Boggian K, Hirzel C, et al. Differences Between Infectious Disease Events in First Liver Transplant Versus Retransplantation in the Swiss Transplant Cohort Study. Liver Transpl. 2021 Sep;27(9):1283–90.

47. Bardou FN, Guillaud O, Erard-Poinsot D, Chambon-Augoyard C, Thimonier E, Vallin M, et al. Tacrolimus exposure after liver transplantation for alcohol-related liver disease: Impact on complications. Transplant Immunology. 2019 Oct;56:101227.

48. Brustia R, Monsel A, Conti F, Savier E, Rousseau G, Perdigao F, et al. Enhanced Recovery in Liver Transplantation: A Feasibility Study. World J Surg. 2019 Jan;43(1):230–41.

49. Angelico R, Gerlach UA, Gunson BK, Neil D, Mergental H, Isaac J, et al. Severe Unresolved Cholestasis Due to Unknown Etiology Leading to Early Allograft Failure Within the First 3 Months of Liver Transplantation. Transplantation. 2018 Aug;102(8):1307–15.

50. Busch CJ, Siegler BH, Werle H, Lichtenstern C, Bruckner T, Heininger A, et al. Risk factors for early viral infections after liver transplantation. Langenbecks Arch Surg. 2018 Jun;403(4):509–19.

51. Ambrus RB, Svendsen LB, Hillingsø JG, Hansen ML, Achiam MP. Post–Endoscopic Retrograde Cholangiopancreaticography complications in liver transplanted patients, a single-center experience. Scand J Surg. 2015 Jun;104(2):86–91.

52. Bodro M, Sabé N, Lladó L, Baliellas C, Niubó J, Castellote J, et al. Prophylaxis versus preemptive therapy for cytomegalovirus disease in high-risk liver transplant recipients: Prevention of CMV Disease in Liver Recipients. Liver Transpl. 2012 Sep;18(9):1093–9.

53. Boudjema K, Camus C, Saliba F, Calmus Y, Salamé E, Pageaux G, et al. Reduced-Dose Tacrolimus with Mycophenolate Mofetil vs. Standard-Dose Tacrolimus in Liver Transplantation: A Randomized Study. American Journal of Transplantation. 2011 May;11(5):965–76.

54. Baccarani U, Isola M, Adani GL, Avellini C, Lorenzin D, Rossetto A, et al. Steatosis of the hepatic graft as a risk factor for post-transplant biliary complications: Graft steatosis and biliary complications. Clinical Transplantation. 2010 Sep;24(5):631–5.

55. Taborelli M, Piselli P, Ettorre GM, Lauro A, Galatioto L, Baccarani U, et al. Risk of virus and non‐virus related malignancies following immunosuppression in a cohort of liver transplant recipients. Italy, 1985–2014. Intl Journal of Cancer. 2018 Oct;143(7):1588–94.

56. Tovikkai C, Charman SC, Praseedom RK, Gimson AE, Van Der Meulen J. Time-varying impact of comorbidities on mortality after liver transplantation: a national cohort study using linked clinical and administrative data. BMJ Open. 2015 May 14;5(5):e006971–e006971.

57. Ojo AO, Leichtman AB, Merion RM. Chronic Renal Failure after Transplantation of a Nonrenal Organ. The New England Journal of Medicine. 2003;

58. Albeldawi M, Aggarwal A, Madhwal S, Cywinski J, Lopez R, Eghtesad B, et al. Cumulative risk of cardiovascular events after orthotopic liver transplantation. Liver Transpl. 2012 Mar;18(3):370–5.

59. AIFA. Linee guida per la compilazione del dossier a supporto della domanda di rimborsabilità e prezzo di un medicinale. 2021.

60. De Simone P, Nevens F, De Carlis L, Metselaar HJ, Beckebaum S, Saliba F, et al. Everolimus With Reduced Tacrolimus Improves Renal Function in De Novo Liver Transplant Recipients: A Randomized Controlled Trial. American Journal of Transplantation. 2012 Nov;12(11):3008–20.

61. Associazione Italiana per lo Studio del Fegato. Raccomandazioni per il trapianto di fegato. 2008.

62. Cecchini I, Monti T, Panza A. Verso una modalità condivisa di profilassi post-OLT con immunoglobuline antiepatite B. BIFE. 2021;
